# Multimodality Diagnostics and Endovascular Large-Bore Aspiration Thrombectomy of the Clot-in-Transit

**DOI:** 10.3390/diagnostics16060917

**Published:** 2026-03-19

**Authors:** Katja Lovoković, Dražen Mlinarević, Vjekoslav Kopačin, Mateo Grigić, Jerko Arambašić, Iva Jurić, Tajana Turk

**Affiliations:** 1 Faculty of Medicine, Josip Juraj Strossmayer University of Osijek, 31000 Osijek, Croatia; 2 Department of Cardiology, University Hospital Centre Osijek, 31000 Osijek, Croatia; 3 Department of Diagnostic and Interventional Radiology, University Hospital Centre Osijek, 31000 Osijek, Croatia

**Keywords:** embolism, pulmonary, percutaneous aspiration thrombectomy, thrombus

## Abstract

Clot-in-transit (CIT) is a free-floating thrombus in the right heart and can enter pulmonary circulation at any moment. Possible treatments include anticoagulation, systemic thrombolysis, surgical embolectomy, and endovascular catheter-based therapies. The optimal treatment is still undetermined, heavily relying on clinical judgment and multidisciplinary team discussion. We report a case of a 70-year-old woman presenting with tachydyspnoea following recent abdominal surgery, who was diagnosed with massive bilateral pulmonary embolism (PE) complicated by a clot-in-transit. Point-of-care ultrasonography revealed a large mobile thrombus in the right atrium with severe right ventricular dysfunction. Due to haemodynamic instability and a contraindication for systemic thrombolysis, mechanical thrombectomy was performed. A large thrombotic burden was aspirated from the right heart and pulmonary arteries, resulting in haemodynamic stabilization and recovery of right ventricular function. The patient remained stable throughout hospitalization and was discharged on oral anticoagulation therapy with complete recovery on follow-up. This case highlights several points. Firstly, CIT is a rare finding but should be considered in patients with massive pulmonary embolism and shock. Furthermore, POCUS is essential for diagnosing CIT. Finally, mechanical thrombectomy is a valuable therapeutic option in high-risk PE patients with contraindications to systemic thrombolysis and haemodynamic instability. Further studies are needed to establish adequate guidelines for the optimal management of CIT patients.

**Figure 1:**
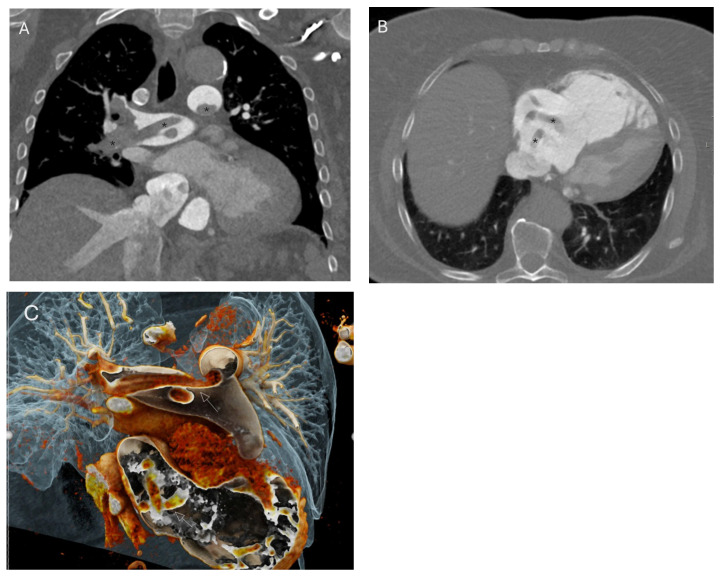
Computed tomography pulmonary angiography (CTPA) demonstrates contrast filling defects (annotated with stars, *) in the left and right pulmonary arteries, interlobar arteries, and the majority of segmental branches, consistent with a massive pulmonary embolism (**A**), as well as a serpiginous filling defect within the right atrium (**B**) consistent with clot-in-transit (CIT). Volume rendering reconstructions (**C**) show serpiginous thrombi in the main pulmonary arteries and right ventricle. CIT is a mobile thrombus temporarily lodged in the right heart, most commonly originating from deep pelvic veins, and can enter the pulmonary circulation at any moment, making it a medical emergency [1]. Therefore, early diagnosis is crucial, especially in haemodynamically unstable patients. The patient was a 70-year-old woman with a history of recent abdominal surgery who presented with acute dyspnoea and obstructive shock. Her blood pressure was 59/29 mmHg, pulse 90 beats per minute, and oxygen saturation (SpO_2_) 98%, with right heart enlargement, suggesting right heart strain.

**Figure 2:**
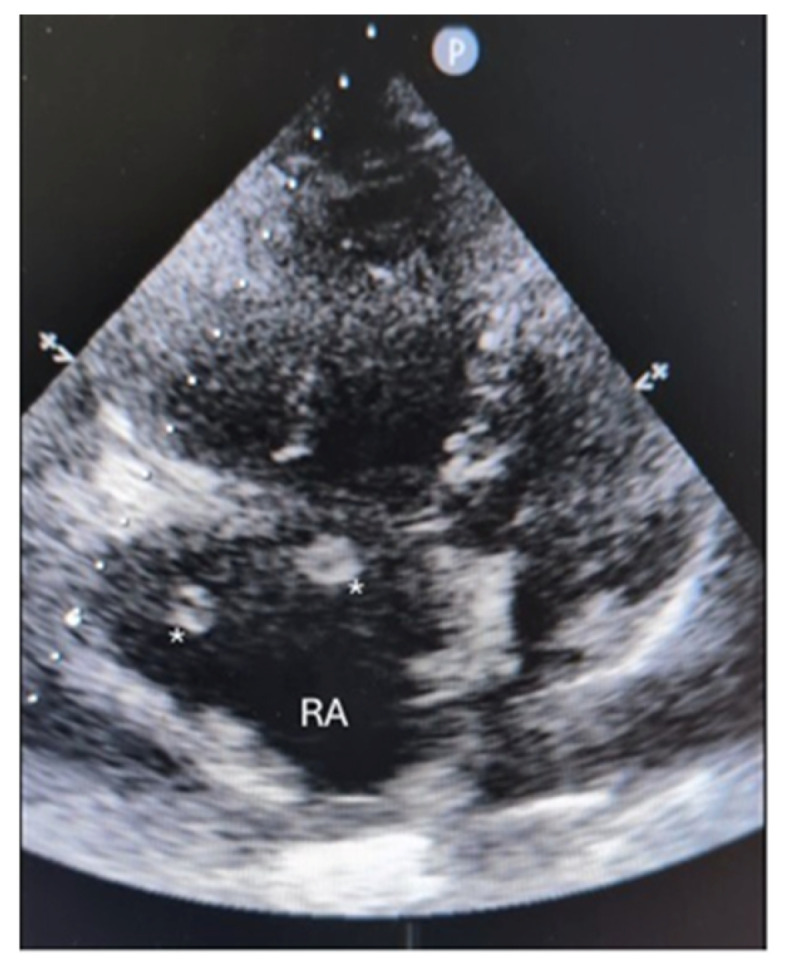
Point-of-care ultrasonography (POCUS) demonstrates a large serpiginous thrombus (annotated with a star, *) in the right atrium (RA), prolapsing through the tricuspid valve into the right ventricle, consistent with a clot-in-transit. POCUS also shows right ventricular dysfunction with a TAPSE of 8 mm and a dilated, plethoric inferior vena cava, supporting the diagnosis of obstructive shock. Although CTPA remains the imaging standard for diagnosing pulmonary embolism, it may not detect intracardiac thrombi due to motion artefacts in tachycardic and dyspnoeic patients, especially if scanning is performed without ECG-synchronized cardiac triggering, with the positive predictive value of CTPA in patients with CIT at approximately 57% [2]. Therefore, POCUS is increasingly used to diagnose CIT and identify pathologies associated with acute PE, such as right heart strain [3]. When performed in patients with acute PE, POCUS can identify CIT in 4% of patients [4]. POCUS is crucial for timely clinical decision-making in diagnoses that require urgent treatment and may not be detected using standard imaging tools and protocols [5].

**Figure 3:**
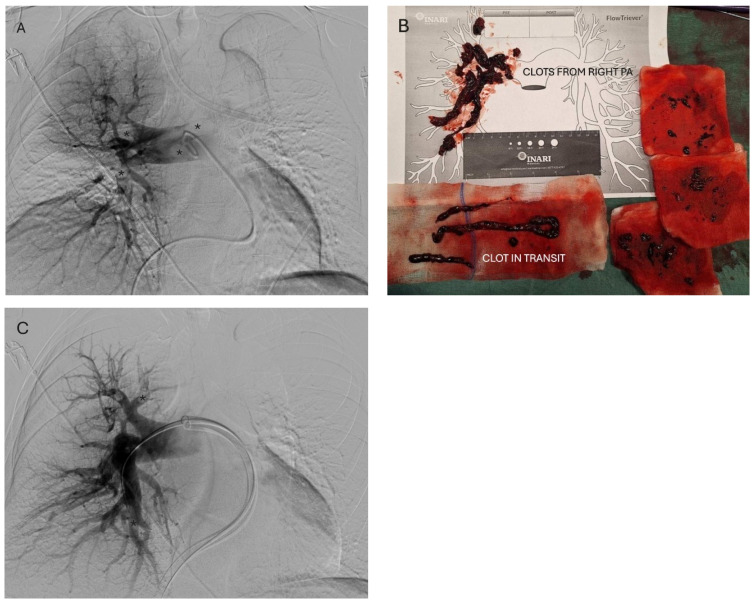
(**A**) Catheter angiography shows contrast filling defects (annotated with stars, *) in the right pulmonary artery branches. According to the 2019 guidelines [6], systemic thrombolysis is the recommended treatment for patients in obstructive shock. However, contraindications to systemic thrombolysis are common in high-risk PE patients, particularly in the postoperative setting. Although these guidelines do not provide recommendations for the treatment of high-risk PE patients with CIT, a recent study suggests that in patients with CIT, systemic thrombolysis or catheter-based treatment, including mechanical thrombectomy, is associated with a significantly lower rate of adverse clinical outcomes compared to anticoagulation alone [7]. Given the patient’s recent abdominal surgery and haemodynamic instability, systemic thrombolysis was contraindicated. After a multidisciplinary team discussion, the decision was made to manage CIT and PE with mechanical thrombectomy, which offers the possibility of rapid reduction in right ventricular strain without added bleeding risk [8]. Under local anesthesia and ultrasound-guided navigation, left common femoral vein puncture was performed, followed by over-the-wire placement of a 24 French introducer sheath (Intri 24, Inari, Irvine, CA, USA). A large-bore aspiration catheter (FlowTriever 24, Inari, Irvine, CA, USA) was advanced over the stiff wire (Amplatz Super Stiff, Boston Scientific, Marlborough, MA, USA) and navigated to the right atrium, where a single aspiration with a dedicated syringe was performed, resulting in incomplete CIT removal. The same aspiration catheter was then navigated to the right pulmonary artery, where six aspirations were performed, evacuating the majority of the right-sided pulmonary thrombus (**B**). Control angiography demonstrated improved filling of the right pulmonary artery branches with only small residual thrombi (**C**). During catheterization of the left pulmonary artery, the patient experienced ventricular tachycardia due to wire manipulation. As the patient’s vital parameters improved—blood pressure 110/60 mmHg, pulse 75 beats per minute, and SpO_2_ 99%—further attempts at thrombectomy were deemed unnecessary. The puncture site was closed with manual compression assisted by a haemostatic sponge. Repeat echocardiography showed normal right atrial and ventricular dimensions, full recovery of right ventricular systolic function (TAPSE 22 mm), and no visible thrombus in the right atrium or pulmonary artery. Throughout the hospital stay, the patient remained haemodynamically stable with no further intervention required.

## Data Availability

The raw data supporting the conclusions of this article will be made available by the authors on request. All figures are original work of the authors.

## Abbreviations

The following abbreviations are used in this manuscript:

CIT	Clot-in-transit
PE	Pulmonary embolism
CTPA	Computed tomography pulmonary angiography
POCUS	Point-of-care ultrasonography
TAPSE	Tricuspid annular plane systolic excursion
USA	United States of America

## References

[1] Igwilo R., Pinsino A., Aksan F., Kapoor S. (2023). Clot-in-transit: A ticking time bomb in the heart with serious consequences. SAGE Open Med. Case Rep..

[2] Mansencal N., Attias D., Caille V., Desperramons J., Guiader J., El Hajjam M., Lacombe P., Nasr I.A., Jardin F., Vieillard-Baron A. (2011). Computed tomography for the detection of free-floating thrombi in the right heart in acute pulmonary embolism. Eur. Radiol..

[3] Reinoso R.E., Leigh Y.O., Salmon J.T., Budde J. (2022). Clot in transit: The role of point-of-care-ultrasound in early diagnosis and improved outcomes. Chest.

[4] Torbicki A., Galié N., Covezzoli A., Rossi E., De Rosa M., Goldhaber S.Z., ICOPER Study Group (2003). Right heart thrombi in pulmonary embolism: Results from the International Cooperative Pulmonary Embolism Registry. J. Am. Coll. Cardiol..

[5] Benser A., Carlson C., Casper E., Ignatowski D., Jain R., Wessly P. (2025). Many Roads Traveled: Sonographer Insights on Clots in Transit—An Echocardiography Case Series. CASE.

[6] Konstantinides S.V., Meyer G., Becattini C., Bueno H., Geersing G.J., Harjola V.P., Huisman M.V., Humbert M., Jennings C.S., Jimenes D. (2020). ESC Scientific Document Group, 2019 ESC Guidelines for the diagnosis and management of acute pulmonary embolism developed in collaboration with the European Respiratory Society (ERS): The Task Force for the diagnosis and management of acute pulmonary embolism of the European Society of Cardiology (ESC). Eur. Heart J..

[7] Zhang R.S., Yuriditsky E., Zhang P., Elbaum L., Bailey E., Maqsood M.H., Postelnicu R., Amoroso N.E., Maldonado T.S., Saric M. (2024). Comparing Management Strategies in Patients with Clot-in-Transit. Circ. Cardiovasc. Interv..

[8] Carroll B.J., Larnard E.A., Pinto D.S., Giri J., Secemsky E.A. (2023). Percutaneous Management of High-Risk Pulmonary Embolism. Circ. Cardiovasc. Interv..

